# Effects of Preparation Methods on the Thermoelectric Performance of SWCNT/Bi_2_Te_3_ Bulk Composites

**DOI:** 10.3390/ma13112636

**Published:** 2020-06-09

**Authors:** Yuqi Liu, Yong Du, Qiufeng Meng, Jiayue Xu, Shirley Z. Shen

**Affiliations:** 1 School of Materials Science and Engineering, Shanghai Institute of Technology, 100 Haiquan Road, Shanghai 201418, China; liuyuqi27@126.com (Y.L.); mengqiufeng@sit.edu.cn (Q.M.); xujiayue@sit.edu.cn (J.X.); 2 CSIRO Manufacturing, Private Bag 10, Clayton South, Melbourne 3169, Australia; shirley.shen@csiro.au

**Keywords:** single-walled carbon nanotube, Bi_2_Te_3_, composite, thermoelectric

## Abstract

Single-walled carbon nanotube (SWCNT)/Bi_2_Te_3_ composite powders were fabricated via a one-step in situ reductive method, and their corresponding bulk composites were prepared by a cold-pressing combing pressureless sintering process or a hot-pressing process. The influences of the preparation methods on the thermoelectric properties of the SWCNT/Bi_2_Te_3_ bulk composites were investigated. All the bulk composites showed negative Seebeck coefficients, indicating n-type conduction. A maximum power factor of 891.6 μWm^−1^K^−2^ at 340 K was achieved for the SWCNT/Bi_2_Te_3_ bulk composites with 0.5 wt % SWCNTs prepared by a hot-pressing process, which was ~5 times higher than that of the bulk composites (167.7 μWm^−1^K^−2^ at 300 K) prepared by a cold-pressing combing pressureless sintering process, and ~23 times higher than that of the bulk composites (38.6 μWm^−1^K^−2^ at 300 K) prepared by a cold-pressing process, mainly due to the enhanced density of the hot-pressed bulk composites.

## 1. Introduction

According to the Seebeck coefficient, thermoelectric (TE) materials can directly convert waste heat into useful electrical energy [1,2]. The TE properties of a material are judged by the figure of merit, *ZT = S^2^σT/κ*, where *S* is the Seebeck coefficient, *σ* is the electrical conductivity, *T* is the absolute temperature, and *κ* is the thermal conductivity [3,4,5,6]. So far, the highest *ZT* value of 2.6 has been reported for SnSe [7]. The actual applied TE devices near room temperature (RT) are mainly made of inorganic bulk materials, e.g., Bi–Te-based alloys due to them being one of the most widely studied TE materials and having exhibited a high *ZT* value at RT [8,9,10]; however, the energy conversion efficiency of TE devices is still much lower than that of the maximum possible Carnot efficiency [2], which has limited the wide application of TE devices. In order to scale out the application of TE devices, the TE performance of inorganic bulk materials should be improved (e.g., optimizing their preparation technologies and compositions), and the conversion efficiency of thermoelectric devices should also be enhanced [11] (e.g., optimizing their geometric structures [12]). 

Carbon nanotubes (CNTs) have extraordinary electrical and mechanical properties [13,14] and can form conductive network structures in a relatively low threshold value when used as fillers for preparation of composite materials; therefore, introducing CNTs into inorganic TE matrixes is an effective method to further improve the TE properties of inorganic materials [15,16,17,18,19,20]. For instance, Choi et al. [15] prepared Te nanowires using a chemical method and then synthesized flexible single-walled carbon nanotube (SWCNT)/Te nanowire films via a vacuum filtering method, and a power factor of 3.40 μWm^−1^K^−2^ was obtained for the composite film with 2 wt % SWCNTs at RT. Zhang et al. [16] first fabricated multi-walled carbon nanotube (MWCNT)/Bi_2_Te_3_ nanocomposite powders via a cryogenic grinding method and then prepared bulk composites through a spark plasma sintering process, and a power factor (PF = *S^2^σ*) of ~800 μWm^−1^K^−2^ (*ZT* value of ~0.3) at 300 K was obtained for the composite with 3 vol% MWCNTs. Ahmad et al. [17] first fabricated SWCNT/Bi_2_Te_3_ nanocomposite powders via a mechanical mixing method and then prepared bulk composites through a high-frequency induction-heated sintering furnace, and a PF of ~900 μWm^−1^K^−2^ (*ZT* value of ~0.32) at 300 K was obtained for the composite with 0.5 wt % SWCNTs. Kim et al. [18] prepared MWCNT/Bi_2_Te_3_ nanocomposite powders via a chemical route combing ball milling method and then fabricated corresponding bulk composites via a spark plasma sintering process, and a PF of 1060 μWm^−1^K^−2^ (*ZT* value of 0.48) at 300 K was achieved for the bulk composite with 0.7 wt % MWCNTs. Hosokawa et al. [19] firstly synthesized Bi_2_Te_3_ nanoplates via a solvothermal method and then prepared Bi_2_Te_3_ nanoplate/SWCNT composite film through a drop-casting technique, and a PF of 91 μWm^−1^K^−2^ for the composite film at 300 K was obtained after an annealing process was applied. Jin et al. [20] fabricated highly ordered Bi_2_Te_3_ nanocrystals anchored on an SWCNT network using a magnetron sputtering technique, and a power factor of ~1600 μWm^−1^K^−2^ (*ZT* value of 0.89) at RT was obtained. However, so far, systematic research of SWCNT/Bi_2_Te_3_ bulk composites using the hydrothermal synthesized SWCNT/Bi_2_Te_3_ nanopowders as raw materials, followed by a cold-pressing combing pressureless sintering process or a hot-pressing process, is limited. Herein, SWCNT/Bi_2_Te_3_ composite powders were fabricated via a one-step in situ reductive method, and their corresponding bulk composites were prepared by a cold-pressing combing pressureless sintering process or a hot-pressing process. The effects of the preparation methods on the composition, microstructure, and TE properties of the bulk composites were investigated.

## 2. Materials and Methods

### 2.1. Materials

Absolute ethanol (C_2_H_5_OH, reagent grade) and bismuth nitrate pentahydrate (Bi(NO_3_)_3_·5H_2_O, analytical reagent (AR)) were purchased from Sinopharm Chemical Reagent Co., Ltd. (Shanghai, China). Potassium borohydride (KBH_4_, AR) and tellurium dioxide (TeO_2_, guaranteed reagent) were purchased from Adamas Reagent Co., Ltd. (Shanghai, China). Sodium tartrate (C_4_H_4_Na_2_O_6_·2H_2_O, ACS reagent) was obtained from Sigma-Aldrich (St. Louis, MO, USA). Potassium hydroxide (KOH, AR) was purchased from General Reagent Co., Ltd. (Qionglai, China). SWCNTs were purchased from Nanjing XFNANO Materials Tech Co., Ltd. (Nanjing, China) All the materials were applied without further purification or treatment.

### 2.2. Preparation of the SWCNT/Bi_2_Te_3_ Composite Powders

**Figure 1:**
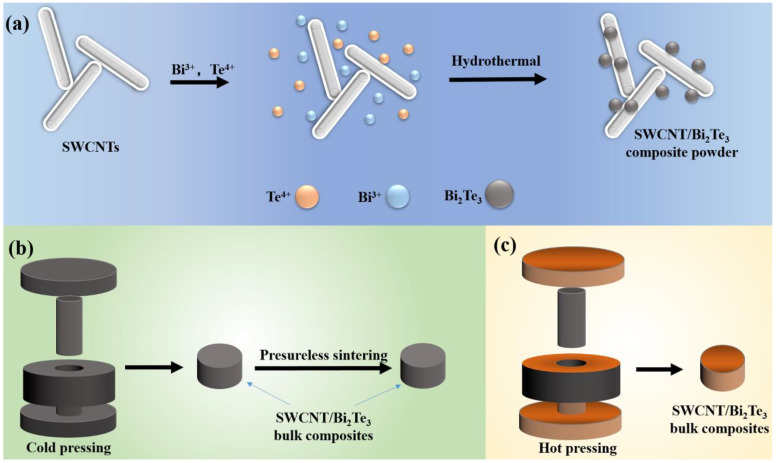
(**a**) The preparation procedures of the single-walled carbon nanotube (SWCNT)/Bi_2_Te_3_ composite powders, (**b**) bulk composites (prepared by a cold-pressing combing pressureless sintering process), and (**c**) bulk composites (prepared by a hot-pressing process).

SWCNT/Bi_2_Te_3_ composite powders were prepared via a one-step in situ reductive method [21]. It mainly contained the following three steps. (1) An appropriate amount of SWCNTs was added in deionized water (80 mL) and then ultrasonicated for 1 h to form Solution I. C_4_H_4_Na_2_O_6_·2H_2_O (2 g), TeO_2_ (0.48 g), and KOH (4.5 g) were added to Solution I and then stirred for 0.5 h to form Solution II. Bi(NO_3_)_3_·5H_2_O (0.97 g) was added into Solution II and then stirred for 2 h to form Solution III. KBH_4_ (1.2 g) was added into Solution III and then stirred for 0.5 h to form Solution IV. (2) Solution IV was transferred to the sealed Teflon-lined autoclave and then heated for 24 h at 180 °C before naturally being cooled to RT. (3) The reaction products were washed several times by deionized water and absolute ethyl alcohol, and then dried at 60 °C for 6 h in a vacuum. The weight percentages (nominal compositions) of SWCNTs in the SWCNT/Bi_2_Te_3_ composite powders were 0.25, 0.5, and 0.75 wt %, respectively. Figure 1a illustrates the preparation procedures of the SWCNT/Bi_2_Te_3_ composite powders.

### 2.3. Preparation of the SWCNT/Bi_2_Te_3_ Bulk Composites

Cold-pressing combing pressureless sintering process: SWCNT/Bi_2_Te_3_ composite powders with different contents of SWCNTs from 0.25 to 0.75 wt % were cold-pressed into pellets at 30 MPa for 0.5 h, and then the bulk composites with 0.5 wt % SWCNTs were pressurelessly sintered at different temperatures (598, 648, or 698 K) for 1 h in an argon (Ar) atmosphere, respectively, before naturally being cooled to RT. The resulting bulk composites are denoted as CNT/BT-PS-598, CNT/BT-PS-648, and CNT/BT-PS-698, respectively. The thicknesses of the as-prepared samples were ~1.3 mm. Figure 1b illustrates the preparation procedures of the SWCNT/Bi_2_Te_3_ bulk composites by a cold-pressing combing pressureless sintering process.

Hot-pressing process: SWCNT/Bi_2_Te_3_ composite powders with 0.5 wt % SWCNTs were hot-pressed into pellets in a graphite die (diameter: 12.7 mm) under vacuum at 80 MPa (648 K) for 1 h, and then naturally cooled to RT. The resulting bulk composites are denoted as CNT/BT-HP-648. The thicknesses of the hot-pressed SWCNT/Bi_2_Te_3_ bulk composites were ~1.55 mm. Figure 1c illustrates the preparation procedures of SWCNT/Bi_2_Te_3_ bulk composites by a hot-pressing process.

### 2.4. Sample Characterization 

The compositions of the as-prepared SWCNT/Bi_2_Te_3_ composites were characterized by X-ray powder diffraction (XRD, D/max 2200PC, Rigaku, Tokyo, Japan). The morphologies of the samples were characterized by high-resolution transmission electron microscopy (HRTEM, Technai G2 F20, FEI, Waltham, MA, USA) and scanning electron microscopy (SEM, Philips PW6800/70, Philips, Amsterdam, The Netherlands). The Seebeck coefficient and electrical conductivity were measured simultaneously from 300 to 360 K by an MRS-3L thin-film thermoelectric test system (Wuhan Giant Instrument Technology Co., Ltd., Wuhan, China) in a low-vacuum atmosphere (≤40 Pa). The instrument test errors are 6% and 5% for the Seebeck coefficient and electrical conductivity, respectively. The thicknesses of the samples were measured by a helical micrometer (Links 150-0.01, Links, Harbin, China). By calculating the ratios of measured density/theoretical density of the SWCNT/Bi_2_Te_3_ bulk materials, the corresponding relative density of the as-prepared sample was obtained. The densities of Bi_2_Te_3_ and SWCNTs used for calculating the theoretical density of the SWCNT/Bi_2_Te_3_ bulk materials were 7.86 [22] and 2.1 g/cm^3^ (from the manufacturer), respectively.

## 3. Results and Discussion

**Figure 2:**
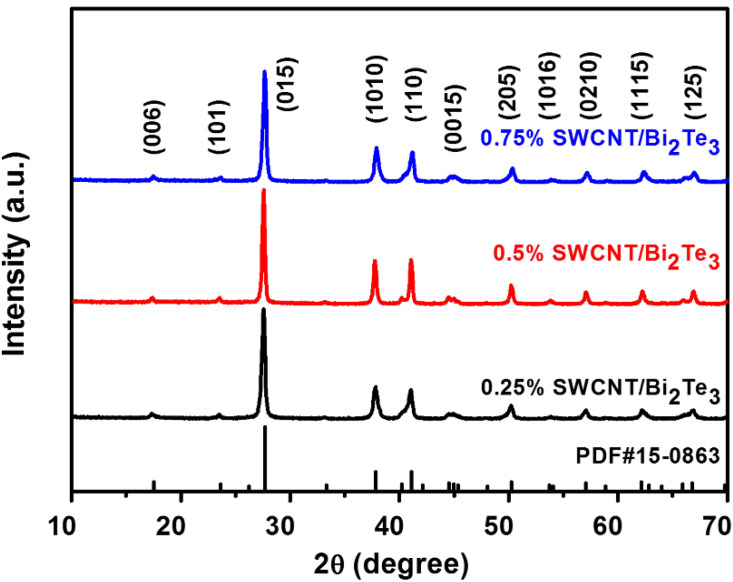
XRD patterns of the SWCNT/Bi_2_Te_3_ composite powders with different contents of SWCNTs from 0.25 to 0.75 wt %.

Figure 2 presents the XRD patterns of SWCNT/Bi_2_Te_3_ composite powders with different contents of SWCNTs from 0.25 to 0.75 wt %. The main diffraction peaks of all the SWCNT/Bi_2_Te_3_ composite powders are matched with the standard JCPDF #15-0863 for Bi_2_Te_3_ [23,24]. No obvious characteristic peak of SWCNTs is observed as the content of SWCNTs increases from 0.25 to 0.75 wt %, mainly due to the low content of SWCNTs, which agrees with a previous report in Reference [17].

**Figure 3:**
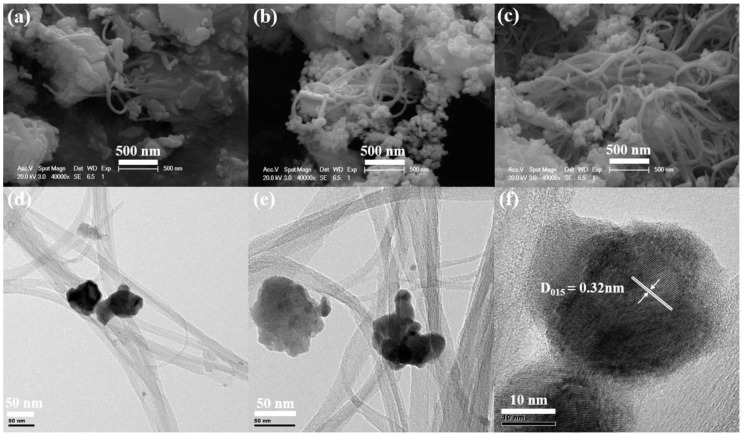
SEM images of the SWCNT/Bi_2_Te_3_ composite powders with different contents of SWCNTs: (**a**) 0.25 wt %, (**b**) 0.5 wt %, and (**c**) 0.75 wt % SWCNTs. TEM images of SWCNT/Bi_2_Te_3_ composite powders with different contents of SWCNTs: (**d**) 0.25 wt %, (**e**) 0.5 wt %, and (**f**) 0.75 wt % SWCNTs.

Figure 3a–c and Figure 3d−f shows the SEM images and TEM images of the SWCNT/Bi_2_Te_3_ composite powders with different contents of SWCNTs, respectively. A network structure is observed for the SWCNTs in the composite powders, which agrees with the results reported in Reference [25] (Figure 3a–c), and some Bi_2_Te_3_ particles are partially nucleated on the surfaces of SWCNTs (Figure 3d–f). The SWCNTs are homogeneously dispersed in the Bi_2_Te_3_ matrixes. From Figure 3f, it can be seen that the lattice spacing is ~0.32 nm, which corresponds to the (015) lattice plane of Bi_2_Te_3_, indicating good crystallinity of the Bi_2_Te_3_ particles in the composite powders [26].

**Figure 4:**
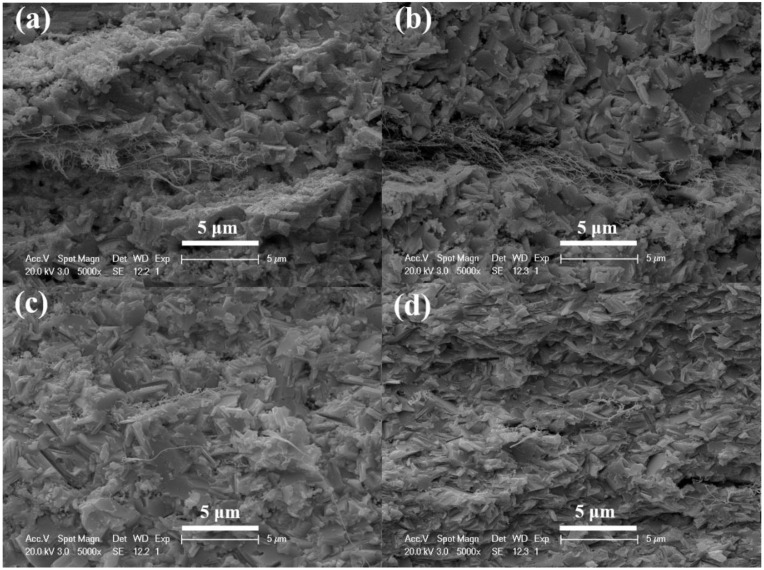
Fracture surface SEM images of (**a**) CNT/BT-PS-598, (**b**) CNT/BT-PS-648, (**c**) CNT/BT-PS-698, and (**d**) CNT/BT-HP-648.

At 300 K, as the content of SWCNTs increases from 0.25 to 0.75 wt %, the *σ* of the cold-pressed SWCNT/Bi_2_Te_3_ bulk composites enhances from 39.5 to 62.9 S/cm, while the absolute values of Seebeck coefficients (*|S|*) are in the range of 70–90 μV/K (the Seebeck coefficients are negative, indicating n-type conduction), and a highest power factor of 38.6 µWm^−1^K^−2^ is obtained for the bulk composites with 0.5 wt % SWCNTs. In order to further enhance the TE properties of the cold-pressed samples, the pressureless sintering and hot-pressing processes were applied for the SWCNT/Bi_2_Te_3_ bulk composites with 0.5 wt % SWCNTs, respectively. Figure 4a–c shows the fracture surface SEM images of CNT/BT-PS-598, CNT/BT-PS-648, and CNT/BT-PS-698, respectively. Figure 4d shows the fracture surface SEM image of CNT/BT-HP-648. It can be seen that the SWCNTs are partly embedded into the Bi_2_Te_3_ matrix, and there are some voids both in the bulk composites prepared by the pressureless sintering process and the hot-pressing process. The content of voids in CNT/BT-HP-648 is much lower than those of CNT/BT-PS-598, CNT/BT-PS-648, and CNT/BT-PS-698, mainly because CNT/BT-HP-648 has undergone a high pressure (80 MPa). An obviously oriented microstructure was observed in CNT/BT-HP-648 (Figure 4d), mainly due to the anisotropy of Bi_2_Te_3_, which agrees with the results reported in Reference [24]. The peak intensity of the (0015) plane (~44.5°) significantly increases in CNT/BT-HP-648 (Figure S1) when compared with the as-prepared composite powders (Figure 2), which also confirms the oriented microstructure of CNT/BT-HP-648. 

**Figure 5:**
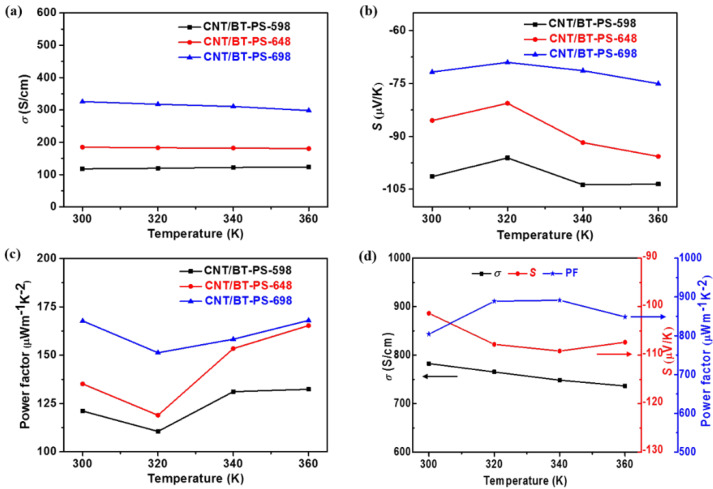
(**a**) Temperature dependence of electrical conductivity, (**b**) Seebeck coefficient, and (**c**) power factors of CNT/BT-PS-598, CNT/BT-PS-648, and CNT/BT-PS-698. (**d**) Temperature dependence of electrical conductivity, Seebeck coefficient, and power factor of CNT/BT-HP-648.

Figure 5a–c shows the TE properties of CNT/BT-PS-598, CNT/BT-PS-648, and CNT/BT-PS-698, as a function of temperature from 300 to 360 K. At 300 K, as the pressureless sintering temperature rises from 598 to 698 K, the *σ* increases from 117.8 to 325.8 S/cm, while the *|S|* decreases from 101.4 to 71.8 μV/K, and a highest power factor of 167.7 µWm^−1^K^−2^ is obtained for the CNT/BT-PS-698, which is ~4 times higher than that of the cold-pressed samples (38.6 µWm^−1^K^−2^ at 300 K) without having undergone pressureless sintering treatment, indicating that pressureless sintering treatment is an effective method to enhance the TE properties of the cold-pressed samples. As the measurement temperature rises from 300 to 360 K, the *σ* of CNT/BT-PS-598, CNT/BT-PS-648, and CNT/BT-PS-698 remains basically unchanged, while the absolute values of the Seebeck coefficient first reduce and then enhance. As a result, the power factors of CNT/BT-PS-598, CNT/BT-PS-648, and CNT/BT-PS-698 first decrease and then increase—for instance, the power factor of CNT/BT-PS-698 decreases from 167.7 (300 K) to 151.3 μWm^−1^K^−2^ (320 K), then it increases to 168.0 μWm^−1^K^−2^ at 360 K. Figure 5d presents the temperature dependence of TE properties of CNT/BT-HP-648 from 300 to 360 K. The *σ* of CNT/BT-HP-648 is 782.6 S/cm at 300 K, which is higher than that of a Bi_2_Te_3_ nanoplate/SWCNT film (111.9 S/cm at 300 K) [19] and that of an SWCNT/Bi_2_Te_3_ bulk material (~700 S/cm at 300 K) [17], while close to that of an MWCNT/Bi_2_Te_3_ bulk (~790 S/cm at 300 K) [18]. The |*S*| of CNT/BT-HP-648 is 101.4 μV/K at 300 K, which is higher than that of a Bi_2_Te_3_ nanoplate/SWCNT composite film (90.1 μV/K at 300 K) [19]; however, it is lower than those of an MWCNT/Bi_2_Te_3_ bulk material (113 µV/K at 300 K) [18] and an SWCNT/Bi_2_Te_3_ bulk material (~115 µV/K at 300 K) [17]. As the measurement temperature rises from 300 to 360 K, the *σ* of CNT/BT-HP-648 shows a decreasing tendency, which suggests the degenerate semiconductor behavior of the hot-pressed bulk composites [27]. The |*S*| of CNT/BT-HP-648 first increases and then decreases in the measurement temperature ranges from 300 to 360 K, and the |*S*| achieves 109.2 μV/K at 340 K. The power factor of CNT/BT-HP-648 first increases and then decreases, due to the same trend of |*S*|, and a highest power factor of 891.6 μWm^−1^K^−2^ is obtained at 340 K. This value (891.6 μWm^−1^K^−2^) is higher than those of the bulk composites prepared by a cold-pressing combing pressureless sintering process (168.0 μWm^−1^K^−2^ at 360 K) and the Bi_2_Te_3_ nanoplate/SWCNT composite film (91 μWm^−1^K^−2^ at 300 K) [19]; however, it is lower than those of the SWCNT/Bi_2_Te_3_ bulk material (~980 μWm^−1^K^−2^ at 400 K) [17] and the MWCNT/Bi_2_Te_3_ bulk material (~1180 μWm^−1^K^−2^ at 423 K) [18].

The relative densities of CNT/BT-PS-598, CNT/BT-PS-648, CNT/BT-PS-698, and CNT/BT-HP-648 are shown in Table 1. As the pressureless sintering temperature rises from 598 to 698 K, the relative densities increase slowly from 78.19% to 81.72%. The relative density of CNT/BT-HP-648 (91.51%) is much higher than those of CNT/BT-PS-598, CNT/BT-PS-648, and CNT/BT-PS-698, which is beneficial to improve the *σ*, as well as the power factor of the as-prepared samples, and it might be the main reason for the higher TE properties of the hot-pressed bulk composites, when compared with the bulk composites prepared by the cold-pressing combing pressureless sintering process. Additionally, it is noted there are still a lot of voids in the bulk composites, which could scatter the phonons and charge carriers, decrease the thermal conductivity, and then improve the *ZT* value of the bulk composites [28].

## 4. Conclusions

SWCNT/Bi_2_Te_3_ bulk composites were prepared by a cold-pressing combing pressureless sintering process or a hot-pressing process, using the hydrothermal synthesized SWCNT/Bi_2_Te_3_ nanopowders as raw materials. For the bulk composites with 0.5 wt % SWCNTs prepared by a cold-pressing combing pressureless sintering process, as the pressureless sintering temperature increased from 598 to 698 K, the electrical conductivity was enhanced, while the absolute value of Seebeck coefficient reduced, and a power factor of 168.0 μWm^−1^K^−2^ at 360 K was obtained, which is much higher than that of the bulk composites (38.6 μWm^−1^K^−2^ at 300 K) prepared by a cold-pressing process; however, it is much lower than that of the bulk composites prepared by a hot-pressing process (891.6 μWm^−1^K^−2^ at 340 K); the main reason is that the hot-pressed SWCNT/Bi_2_Te_3_ bulk composites have much higher relative densities. To this end, the hot-pressing process is a better choice than the cold-pressing or cold-pressing combing pressureless sintering processes, although it needs expensive equipment. The TE performance of the SWCNT/Bi_2_Te_3_ bulk composites could be further improved by enhancing their relative densities via optimizing the parameters for the hot-pressing process, or controlling the parameters of the gradient cooling process for the cold-pressing combing pressureless sintering process. The as-prepared SWCNT/Bi_2_Te_3_ bulk composites have a potential for thermoelectric devices.

## Figures and Tables

**Table 1:** Relative densities of the as-prepared bulk composites.

Samples	Relative Densities (%)
CNT/BT-PS-598 CNT/BT-PS-648 CNT/BT-PS-698 CNT/BT-HP-648	78.19 79.53 81.72 91.51

## Supplementary Materials

The following are available online at https://www.mdpi.com/1996-1944/13/11/2636/s1, Figure S1: XRD patterns of the CNT/BT-HP-648.

## References

[1] Zhang X., Zhao L.-D. (2015). Thermoelectric materials: Energy conversion between heat and electricity. J. Mater..

[2] Du Y., Xu J., Paul B., Eklund P. (2018). Flexible thermoelectric materials and devices. Appl. Mater. Today.

[3] Hung N.T., Hasdeo E.H., Nugraha A.R.T., Dresselhaus M.S., Saito R. (2016). Quantum effects in the thermoelectric power factor of low-dimensional semiconductors. Phys. Rev. Lett..

[4] Ibanez M., Luo Z., Genç A., Piveteau L., Ortega S., Cadavid D., Dobrozhan O., Liu Y., Nachtegaal M., Zebarjadi M. (2016). High-performance thermoelectric nanocomposites from nanocrystal building blocks. Nat. Commun..

[5] Poudel B., Hao Q., Ma Y., Lan Y., Minnich A., Yu B., Yan X., Wang D., Muto A., Vashaee D. (2008). High-thermoelectric performance of nanostructured bismuth antimony telluride bulk alloys. Science.

[6] Chang C., Wu M., He D., Pei Y., Wu C.-F., Wu X.-F., Yu H., Zhu F., Wang K., Chen Y. (2018). 3D charge and 2D phonon transports leading to high out-of-planeZTin n-type SnSe crystals. Science.

[7] Zhao L.-D., Lo S.-H., Zhang Y., Sun H., Tan G., Uher C., Wolverton C., Dravid V.P., Kanatzidis M.G. (2014). Ultralow thermal conductivity and high thermoelectric figure of merit in SnSe crystals. Nature.

[8] Jabar B., Qin X.Y., Li D., Zhang J., Mansoor A., Xin H.X., Song C.J., Huang L.L. (2019). Achieving high thermoelectric performance through constructing coherent interfaces and building interface potential barriers in n-type Bi_2_Te_3_/Bi_2_Te_2.7_Se_0.3_ nanocomposites. J. Mater. Chem. A.

[9] Xie W.-J., He J., Kang H.J., Tang X., Zhu S., Laver M., Wang S., Copley J.R.D., Brown C.M., Zhang Q. (2010). Identifying the specific nanostructures responsible for the high thermoelectric performance of (Bi,Sb)_2_Te_3_ nanocomposites. Nano Lett..

[10] Snyder G.J., Toberer E.S. (2008). Complex thermoelectric materials. Nat. Mater..

[11] Bell L.E. (2008). Cooling, heating, generating power, and recovering waste heat with thermoelectric systems. Science.

[12] Du Y., Chen J.G., Meng Q.F., Dou Y.C., Xu J.Y., Shen S.Z. (2020). Thermoelectric materials and devices fabricated by additive manufacturing. Vacuum.

[13] Choi J., Lee J.Y., Lee H., Park C.R., Kim H. (2014). Enhanced thermoelectric properties of the flexible tellurium nanowire film hybridized with single-walled carbon nanotube. Synth. Met..

[14] Yao Q., Chen L., Zhang W., Liufu S., Chen X. (2010). Enhanced thermoelectric performance of single-walled carbon nanotubes/polyaniline hybrid nanocomposites. ACS Nano.

[15] Choi J., Lee K., Park C.R., Kim H. (2015). Enhanced thermopower in flexible tellurium nanowire films doped using single-walled carbon nanotubes with a rationally designed work function. Carbon.

[16] Zhang Q., Xu L., Zhou Z., Wang L., Jiang W., Chen L. (2017). Constructing nanoporous carbon nanotubes/Bi_2_Te_3_ composite for synchronous regulation of the electrical and thermal performances. J. Appl. Phys..

[17] Ahmad K., Wan C. (2017). Enhanced thermoelectric performance of Bi_2_Te_3_ through uniform dispersion of single wall carbon nanotubes. Nanotechnology.

[18] Kim K.-T., Choi S.-Y., Shin E.H., Moon K.-S., Koo H.Y., Lee G.-G., Ha G.H. (2013). The influence of CNTs on the thermoelectric properties of a CNT/Bi2Te3 composite. Carbon.

[19] Hosokawa Y., Takashiri M. (2019). Impact of the amount of single-wall carbon nanotubes (SWCNTs) in single-crystalline Bi_2_Te_3_ nanoplates/SWCNTs nanocomposite films by drop-casting method. Jpn. J. Appl. Phys..

[20] Jin Q., Jiang S., Zhao Y., Wang N., Qiu J., Tang D., Tan J., Sun D.-M., Hou P.-X., Chen X.-Q. (2018). Flexible layer-structured Bi2Te3 thermoelectric on a carbon nanotube scaffold. Nat. Mater..

[21] Du Y., Li J., Xu J., Eklund P. (2019). Thermoelectric properties of reduced graphene Oxide/Bi_2_Te_3_ nanocomposites. Energies.

[22] Liang B., Song Z., Wang M., Wang L., Jiang W. (2013). Fabrication and thermoelectric properties of graphene/Bi_2_Te_3_ composite materials. J. Nanomater..

[23] Kumar S., Singh S., Dhawan P.K., Yadav R.R., Khare N. (2018). Effect of graphene nanofillers on the enhanced thermoelectric properties of Bi_2_Te_3_ nanosheets: Elucidating the role of interface in de-coupling the electrical and thermal characteristics. Nanotechnology.

[24] Du Y., Cai K., Li H., An B.J. (2010). The influence of sintering temperature on the microstructure and thermoelectric properties of n-type Bi_2_Te_3−x_Se_x_ nanomaterials. J. Electron. Mater..

[25] Du Y., Shen S., Yang W., Cai K., Casey P. (2012). Preparation and characterization of multiwalled carbon nanotube/poly(3-hexylthiophene) thermoelectric composite materials. Synth. Met..

[26] Zhou L., Zhang X., Zhao X., Sun C., Niu Q. (2010). Synthesis and characterization of carbon nanotube supported Bi_2_Te_3_ nanocrystals. J. Alloy. Compd..

[27] Lee G.-E., Kim I.-H., Choi S.-M., Lim Y.S., Seo W.-S., Park J.-S., Yang S.-H. (2014). Process controls for Bi_2_Te_3_-Sb_2_Te_3_ prepared by mechanical alloying and hot pressing. J. Korean Phys. Soc..

[28] Du Y., Chen J., Meng Q., Xu J., Paul B., Eklund P. (2020). Flexible ternary carbon black/Bi_2_Te_3_ based alloy/polylactic acid thermoelectric composites fabricated by additive manufacturing. J. Mater..

