# Supplementary material for: Effects of Preparation Methods on the Thermoelectric Performance of SWCNT/Bi2Te3 Bulk Composites
Source: Materials (Basel). 2020 Jun 9;13(11):2636. doi: 10.3390/ma13112636 (PMC7321600; doi:10.3390/ma13112636)
Supplement: Supplementary file 1 [file materials-13-02636-s001.pdf]

materials

MDPI

Supplementary Materials

Effects of Preparation Methods on the Thermoelectric Performance of SWCNT/Bi_2Te_3 Bulk Composites

Yuqi Liu [1], Yong Du [1,*], Qiufeng Meng [1], Jiayue Xu [1] and Shirley Z. Shen [2]

[1] School of Materials Science and Engineering, Shanghai Institute of Technology, 100 Haiquan Road, Shanghai 201418, China; liuyuqi27@126.com (Y.L.); mengqiufeng@sit.edu.cn (Q.M.); xujiayue@sit.edu.cn (J.X.)

[2] CSIRO Manufacturing, Private Bag 10, Clayton South, Melbourne 3169, Australia; shirley.shen@csiro.au

* Correspondence: ydu@sit.edu.cn; Tel.: +86-1821-701-7450

Received: 29 April 2020; Accepted: 4 June 2020; Published: date

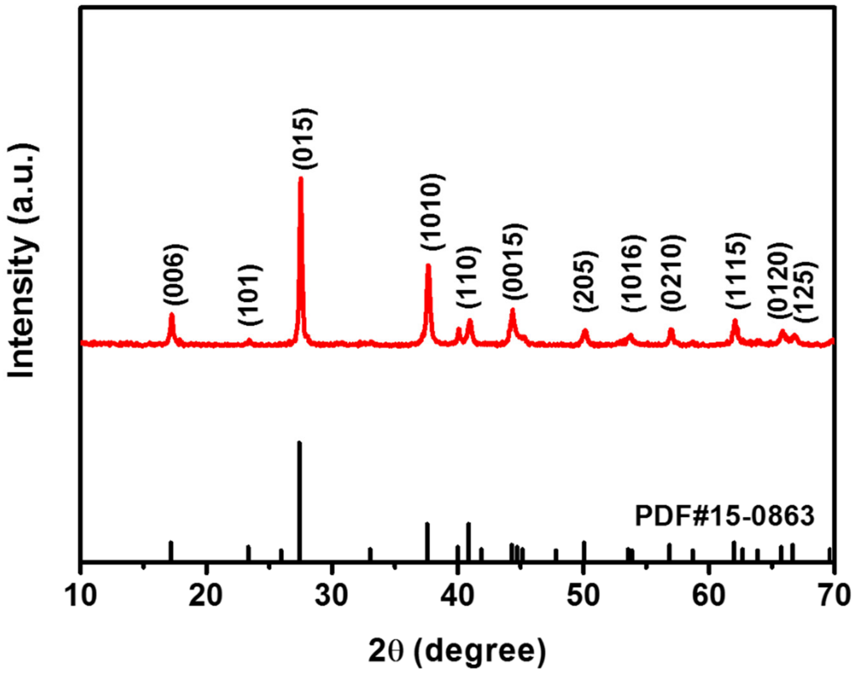

Figure S1. XRD patterns of the CNT/BT-HP-648.

© 2020 by the authors. Submitted for possible open access publication under the terms and conditions of the Creative Commons Attribution (CC BY) license (http://creativecommons.org/licenses/by/4.0/).
